# Validating the Data Completeness and Accuracy of the Canadian Cystic Fibrosis Registry

**DOI:** 10.1155/carj/8893074

**Published:** 2025-07-01

**Authors:** Ranjani Somayaji, Stephanie Y. Cheng, Noma Abdulrahem, Sanja Stanojevic, Paul Eckford, Bradley S. Quon, Elizabeth Cromwell, Albert Faro, Christopher H. Goss, Anne L. Stephenson

**Affiliations:** ^1^Department of Medicine, Cumming School of Medicine, University of Calgary, Calgary, Canada; ^2^Cystic Fibrosis Canada, Toronto, Canada; ^3^Department of Community Health and Epidemiology, Dalhousie University, Halifax, Nova Scotia, Canada; ^4^Department of Medicine, University of British Columbia, Vancouver, Canada; ^5^Cystic Fibrosis Foundation, Bethesda, Maryland, USA; ^6^Division of Pulmonary, Critical Care and Sleep Medicine, Department of Medicine, University of Washington, Seattle, Washington, USA; ^7^Division of Pulmonary and Sleep Medicine, Department of Pediatrics, University of Washington, Seattle, Washington, USA; ^8^Division of Respirology, St. Michael's Hospital, University of Toronto, Toronto, Ontario, Canada

## Abstract

**Introduction:** The Canadian Cystic Fibrosis Registry (CCFR) was developed in the 1970s and has longitudinal demographic and clinical data on persons living with cystic fibrosis (CF) attending accredited clinics in Canada. We aimed to validate the data collection and identify potential limitations of the CCFR.

**Methods:** Of 40 accredited CF clinics in Canada invited and based on an a priori sample size calculation, eight clinics were included. 15% of each CF clinic's population in 2019 were randomly selected. Data variables were selected based on their importance to care, epidemiologic trends, and data related to demography, clinic visits, and hospitalizations. The accuracy of the registry data was compared to the medical records as the gold standard. Each data element was categorized as correct, incorrect, or not able to be validated. The accuracy rate was calculated as the percent correct out of all records validated.

**Results:** A total of 4382 individuals had data entered into the CCFR in 2019. The validation cohort consisted of 208 individuals from 8 clinics, which were representative across location, size of clinic (small/medium/large), and type of clinic (adult, pediatric, and combined). The 208 individuals were 52% male and 95% White, and with a median age of 26.3 years (IQR: 15.2–36.6). Approximately 95% of CCFR data on clinical measurements, infections, treatments, and hospitalizations validated were accurate as compared to the medical record. For demography, sex and date of birth had 100% accuracy.

**Conclusion:** Our validation of the CCFR demonstrated high accuracy for clinical and demographic variables used in clinical research.

## 1. Introduction

Cystic fibrosis (CF) is a genetic multisystem disease with an incidence of approximately one per 3848 live births in Canada and an estimated 80 new diagnoses annually [1, 2]. Impressively, in Canada in 2022, the median survival in people with CF (pwCF) has reached 60 years in contrast to two decades ago when it was 37 years of age based on the Canadian CF Registry (CCFR) data and speaks of the advancements in research and care in this field [1].

Initially, the CCFR was an extension of the United States' CF Foundation (CFF) Patient Data Registry whose cornerstone was laid by Dr. Warwick in 1966. By 1973, Canadian CF clinics (*N* = 20) were contributing Canadian data to the United States' CFF Registry and the first formal joint United States–Canadian CF report summarizing data captured from 1966 to 1976 that was published in 1978 [3]. This report detailed information on national CF birth and death rates, survival curves, and age of diagnosis. In the late 1970s, clinical data such as lung function and markers of nutrition (e.g., height and weight) and microbiology were added to the registry. In 1984, CF Canada established a standalone CCFR that included all historical data dating back to the 1970s and continues to capture patient demographic and clinical and vital statistics longitudinally across the lifetime of the individual.

Currently, all accredited CF clinics are supported by clinic incentive grants contingent on data submission to the CCFR, and each clinic is uniquely able to access aggregate data relating to national epidemiologic trends as well as patient-level data pertaining to their respective patient population. Incentives for pwCF to receive care from Canadian CF centers and hence have their data captured in the registry include being able to obtain medications from a pharmacy associated with a CF clinic where many of their CF medications are paid for by provincial drug plans. It is estimated that less than 1% of individuals who are approached to participate in the CCFR decline consent to participate (personal communication, CF Canada), which enables powerful population-based analyses as well as access by pwCF to research trials and quality improvement initiatives [4].

Given the fact that the CCFR is a valuable tool in clinical care, surveillance of epidemiologic trends, and research, much effort has gone into iterative updates and improvements in data collection and monitoring. The CCFR data can be used by care teams in their clinical interactions, planning for needed resources, and understanding their practice in the national context. Each year, an Annual Data Report is published by CF Canada which documents key demographic and clinical metrics to paint a picture of the evolving landscape [1]. With the large therapeutic pipelines in CF, the CCFR plays an important role in the provision of pharmaceutical postapproval data for regulators as well as obtaining an understanding of real-world effectiveness [5]. To obtain CCFR data, researchers are able to submit an application to CF Canada, which is then reviewed by a panel of CF researchers for approval [6], and these embedded record-level case-report data may be used for retrospective or prospective analyses.

Leveraging population-based registries for research and clinical purposes requires that the data integrity is of the highest quality. In the United States, Knapp et al. conducted an audit of the CFF Patient Registry (CFFPR) and demonstrated that the data reported in the registry had a high agreement with the patient's medical charts [7]. Although CF Canada strives to ensure the same with the CCFR, no formal validation of the data has been previously conducted. Therefore, the aim of this study was to conduct an audit of the accuracy of the data captured within the CCFR. The hypothesis was that the data would be highly accurate and complete for key demographic and clinical metrics.

## 2. Methods

### 2.1. Study Population

All consenting persons who are diagnosed with CF attending an accredited CF clinic in Canada, who consented to participate in the CCFR and therefore have data in the CCFR, were eligible for the study. CF Canada maintains a network of 40 accredited pediatric and/or adult clinics across Canada. Each CF clinic program obtains informed consent or assent from patient participants or guardians. CF Canada provides guidelines, training, and support for data entry into the CCFR and serves as a coordinating hub for data collection and analysis. The study was approved by the Research Ethics Board at St. Michael's Hospital in Toronto, Ontario (REB22-085), the University of Calgary (REB22-0380), and covered by the preexisting ethics for CCFR collection and use at each of the Canadian CF centers.

### 2.2. CCFR Data Collection

Data are collected through a secure, web-based portal containing demographic, diagnosis, clinical encounters, care episodes, and annual review forms. Data entry is completed by a CF clinic staff member through information obtained by medical assessment and review of the medical records. The demographic form consists of information regarding date of birth, sex, race, and place (province/country) of birth, as well as date and cause of death, if applicable. The diagnosis form captures information on the date of diagnosis, signs or symptoms leading to a diagnosis, and results of diagnostic tests (e.g., sweat chloride and genotype). Encounter forms collect data relating to clinic visit dates, anthropometric information, medications (acute and chronic), airway microbiology (culture type/results), pulmonary function tests, complications, and laboratory tests. Care episodes capture hospitalizations and home treatments and their reasons (e.g., intravenous [IV] antibiotics). Each year, CF clinics are also asked to collect information on socioeconomic and other data variables not captured in other forms, such as marital status, pregnancy details, employment, and vaccinations. Transplantation is captured in a separate form with detailed information on the transplant journey (referral, listing, and receipt of transplant) and the organ transplanted. The CCFR is set up such that different clinics that follow the same patient can enter data into a single profile and also ensure that all pertinent data are available to any provider.

### 2.3. Registry Data Validation

In collaboration with members of the CF Canada Registry team, we initiated a data validation program to assess the completeness and accuracy of critical fields of the 2019 CCFR in comparison to the medical record (paper chart or electronic). The 2019 data were the most recent year expected to have data completeness prior to the SARS-CoV-2 pandemic. A convenience sample of eight clinics (covering 15% of the patient population) was included in the audit. Eight clinics that were representative by size (small/medium/large), coverage (adults/pediatric), and geography agreed to participate. Clinic size was based on the tertiles of the number of individuals reported in the CCFR in 2019: large (> 128), medium (64–127), and small (≤ 63). A minimum of 20 and a maximum of 50 patients for each clinic were randomly selected, with a stratified sampling strategy used to ensure a representation of clinically important CF-related complications: CF-related diabetes and CF liver disease. An a priori sample size calculation determined that 160 patient charts would need to be accessed to assess completeness and accuracy while ensuring the error rate was less than our assigned acceptable limit of 10% at a Type II error rate of 5%. At each clinic, the data validation was conducted by a staff member who received standardized training and secured materials via ShareFile for the work. The staff member who conducted the validation was not part of the clinical care team who routinely entered data into the CCFR.

For the data validation, data variables were selected based on their importance to patient care and after assessing overall care trends and outcomes in CF. The data validation database was developed as a Microsoft Excel spreadsheet containing the necessary information from the CCFR for the fields to be evaluated and presented in four tabs: (1) consent, (2) patient data, (3) annual data, and (4) transplant data. Consent forms were assessed first and if found to be missing, incorrect, or incomplete, the audit of the particular individual was stopped. If a patient had more than 5 clinical measurements for longitudinal data (e.g., lung function), five were randomly selected for the audit.

The audit cohort was descriptively summarized alongside the full CCFR cohort of 2019. The medical record was presumed to be the gold standard. Further details regarding the audit template and variable list are provided in the Supporting Information section (Appendix A). Completeness of the data variables was assessed with the number of total records accessed and missingness recorded. For continuous variables (e.g., height, weight, and lung function), a value was considered accurate if it was within 1% of the value in the medical record. For date variables, a value was considered accurate if the month and year were the same as the medical record. If sufficient information was not available in the medical record for a given variable to assess accuracy, this was classified as “could not be validated.” If the information was missing in the CF Registry (for example, lung function measurements for children under 6 who do not routinely undergo this test), it was classified as “missing.” The number of records validated was calculated as the total number of records less the records that could not be validated or were considered missing. Accuracy was defined as the proportion of cases whereby the CCFR and medical record data matched and there were available data in both. The proportion of correct and incorrect data points, as calculated by dividing the number of matched and unmatched cases, respectively, by the number of records, was validated.

## 3. Results

### 3.1. CCFR CF Clinic Characteristics

There were a total of 40 clinic programs across Canada with a distribution of pediatric, adult, and combined pediatric and adult clinics of 40%, 42.5%, and 17.5%, respectively. Of the clinics, 80% were English-speaking and were distributed equally by thirds into small, medium, and large sizes. Among the 8 CF clinics selected for data validation (20% of the total number of CF clinics), the clinic type was distributed as follows: pediatric (50%), adult (37.5%), and combined pediatric and adult (12.5%). 87.5% were English-speaking clinics and there was a slight overrepresentation of small-sized CF clinics (50%) (Table 1).

### 3.2. CCFR CF Patient Characteristics

In 2019, there were a total of 4382 individuals diagnosed with CF whose data were entered into the CCFR (Table 1). Of these, 2351 (53.7%) were male and 4073 (92.5%) were White and had a median age of 23.7 years (interquartile range [IQR]: 11.9–35.5). Among these individuals, 327 (7.5%) had undergone a lung transplant. A total of 208 CCFR patient records (4.7% of the CF patients in the CCFR in 2019) were reviewed for the data validation and all had consent in place. They consisted of 109 (52.4%) males, 198 (95.2%) were white, and had a median age of 26.3 years (IQR: 15.2–36.6). Of the validation cohort, 28 (13.5%) had undergone a lung transplant procedure (Table 1).

### 3.3. Data Quality

The 208 patients had a total of 693 sets of clinical measurements (e.g., weight, height, and lung function) and 194 hospitalizations reviewed in the 2019 audit. Overall, over 97% of the clinical measurements and over 98% of the hospitalizations were correctly entered in the CCFR. The data entered into the CCFR matched the medical record in 92.3%–100% of instances depending on the variable (Table 2). Date of birth and biological sex had 100% concordance between the CCFR and medical records. All medications, including inhaled antibiotics, in the CCFR matched the medical record in 92.7%–100.0% of cases. CF-related complications were entered with a high level of accuracy (> 98%). The lowest level of accuracy was found with the date of diagnosis (92.9%) and date of transplant (92.3%) with the remainder of the variables all matching in more than 95% of instances (Table 2).

## 4. Discussion

As a recognized example of a successful population-based registry, the CCFR comprises the vast majority of pwCF attending accredited CF clinics. In our validation of the CCFR data, we identified that the registry data are complete and accurate for meaningful clinical variables with little missingness. Of the Canadian population included in the registry, we identified that clinical measurements and hospitalizations were accurate in approximately 96% or more of cases. Biological sex (at birth) and date of birth were concordant between the CCFR and medical records in all cases. Medications and CF-related complications were also entered with high accuracy in more than 90% of cases. The lowest accuracy in the medications category was for pancreatic enzymes use, which may be due to how data are entered, since the variable indicating pancreatic status (pancreatic insufficiency vs. sufficiency) is entered as a separate field and matched in 99.0% of cases. This speaks about the need to consider challenges with accuracy when variables with overlap or redundancy are collected. Notably, newer therapies such as CFTR modulators inclusive of start dates were entered with complete accuracy. This is of particular importance as the registry can be used to identify those eligible for clinical trials of novel therapeutics and requires that these data be accurate.

With the evolution of registries and their interfaces (i.e., online portals) such as the CCFR, quality improvement initiatives have become an increasingly important focus and more feasible at the population level. The successful use of the United States-based CFFPR for individual, clinic, and national level initiatives as well as serving as a repository of quality improvement resources has led to the adoption of this model across multiple countries. As an example, Germany developed a quality improvement program enabling the identification of approaches that can lead to improved CF outcomes [8, 9]. The comparison of nutritional outcomes in the Toronto and Boston CF centers is yet another example of the power of a proactive quality improvement program [10], as these results led to significant shifts in nutritional interventions for pwCF. The SARS-CoV-2 pandemic also showcased the ability of multicountry registries to harmonize and identify the impacts of the pandemic on pwCF [11]. There is a completed and ongoing work to assess differing care models, treatment approaches, and their resulting outcomes demonstrating the power that can be harnessed from population-based registries [12–15]. Furthermore, efforts are underway to link CCFR data with other sources which may further the utility of the registry and serve as another quality assurance strategy [16, 17].

Other countries with established CF registries report coverage ranging from 41% to 99% of their CF population and may also vary in the breadth and design of data collection such as whether the data are collected at each encounter or only annually [18]. The Knapp et al.'s audit of the United States' CF registry estimated that the CFFPR includes approximately 81%–84% of pwCF in the country on the basis of their attendance at an accredited center, where the estimate of individuals is not yet diagnosed as well as the consent for the registry [7, 19]. The United States' CFFPR audit identified that they had a similar high accuracy of clinic visits and hospitalizations. A French CF registry audit undertaken by Pellen et al. demonstrated that there was a high degree of accuracy in clinical measures, but variability was noted in the measurement processes such as for lung function (based on the use of different reference equations) between clinical centers [20]. As there have increasingly been harmonized analyses to examine important outcomes relating to lung health and survival [21, 22], it is important to ensure that the registries are sufficiently similar in their accuracy and documentation on variable definitions to ensure appropriate harmonization of data across registries [23].

Although a number of key clinical variables relating to health encounters and medications were accurately recorded in the CCFR, variables such as date of diagnosis and race were less accurate. Challenges with validating the date of CF diagnosis may reflect the loss of these precise data as individuals transition from pediatric to adult clinics and may be recorded as an early or late diagnosis in adulthood. It may be that the date of diagnosis has a limited utility and predictive value or impact in an audit rather than having a general timeframe of diagnosis (i.e., infancy, early childhood, and adult). In our validation, race had a high level of accuracy (99.4%); however, it also has a large number of records that could not be validated (28). This variable is often determined by the CF clinic staff rather than reported by the patients themselves and may not accurately reflect the true diversity of the CF population. Furthermore, the categories used to define race are not comprehensive and may not reflect multiracial backgrounds accurately. Evaluations in other large observational health datasets such as the United States-based Healthcare Costs and Utilization Project have demonstrated that race or ethnicity was unknown in 57% of patients (of 160 million), and information was discrepant with the electronic health record in two-thirds of cases [24]. Race has long been collected in datasets for administrative, clinical, and research purposes. However, there are a myriad of issues to its collection, not the least of which is that race is a social construct without a clear correlation to ancestry [25, 26], and compounded by issues of cultural insensitivity and lack of understanding relating to its use and risks for discrimination [27–30]. To work toward diminishing health inequities [31], employing multifaceted approaches inclusive of patient engagement and adopting an antiracism culture may be key to better collection of self-identified ethnicity/ancestry data more compassionately and accurately [26].

Our validation had little to no missingness in the CCFR data with the exception of lung function values (110 records, 16% of the total number of records). However, it is important to note that lung function is not typically measured in children < 6 years of age. Young children will have height and weight measured during a clinic visit but their lung function (i.e., ppFEV_1_) would be recorded as missing for the purpose of data validation. Although we made efforts to obtain a representative sample of CF clinics by geographic region and clinic size for the audit, there was overrepresentation by pediatrics, smaller clinic sizes, and English-speaking clinics. However, among the individuals with CF who were included in the data validation, they appeared to be representative of the general Canadian CF population, with a slight overrepresentation of those living posttransplant. Moreover, we cannot be certain that the full Canadian picture is represented by our sample although we used a stratified random sampling strategy within clinics and there are national standards for clinical practice which would minimize this bias. We made an assumption about the accuracy of the clinical record, but it is possible that inaccuracies from the medical chart were transcribed into the registry when original documents were not available. Given the cross-sectional nature of our audit, longitudinal trends or loss to follow-up were not systematically evaluated; however, the loss to follow-up rates are low (2.5%) as reported in prior publications [21] and in annual CF registry reports [1]. Our variable selection was broadly consistent with that of Knapp et al. in the 2016 CFFPR audit enabling comparison [7], but future data validation projects could include preventative measures such as vaccines, as well as pregnancy data, as these may be important health metrics to consider. Despite the limitations, our national validation of the CCFR demonstrated a high degree of accuracy and completeness and further reinforces the importance of population-based registries for advancing clinical care.

## 5. Conclusion

In summary, our validation of the CCFR identified that key demographic and clinical metrics were highly accurate and complete when compared to the medical chart. The inaccuracies in the date of diagnosis are likely related to archiving or loss of medical records as individuals transition from pediatric to adult centers and may ultimately be of less importance as compared to the general timeframe of diagnosis. Race is a social construct with limited correlation to ancestry, and inaccuracies in its collection may be representative of a complex problem that requires thoughtful, multifaceted approaches to improve self-reported collection relating to ethnicity. The study reinforces the role of the CCFR as an important resource for research, clinical care, and quality improvement for pwCF living in Canada.

## Figures and Tables

**Table 1:** Comparison of validated CF clinics and Canadian CF clinics.

	Canadian CF clinics	Validated CF clinics
Characteristics of the CF clinic		
Number of clinics	40	8
CF clinic size^∗^		
Large	13 (32.5)	3 (37.5)
Medium	14 (35.0)	1 (12.5)
Small	13 (32.5)	4 (50.0)
Type of clinic		
Pediatric	16 (40.0)	4 (50.0)
Adult	17 (42.5)	3 (37.5)
Combined	7 (17.5)	1 (12.5)
Language of clinic^†^		
English-speaking	32 (80.0)	7 (87.5)
French-speaking	8 (20.0)	1 (12.5)
Region of location^‡^		
West	13 (32.5)	2 (25.0)
Ontario	12 (30.0)	1 (12.5)
Quebec	10 (25.0)	1 (12.5)
East	5 (12.5)	4 (50.0)
Characteristics of individuals with CF		
Number of individuals	4382	208
Age, median (IQR)	23.7 (11.9–35.5)	26.3 (15.2–36.6)
Sex		
Female	2031 (46.4)	99 (47.6)
Male	2351 (53.7)	109 (52.4)
Race		
White	4073 (92.5)	198 (95.2)
Non-White^§^	309 (7.1)	10 (4.8)
Postlung transplant	327 (7.5)	28 (13.5)

*Note:* Values are *N* (%) unless otherwise stated.

^∗^Clinic size based on tertiles of the number of individuals reported in the CCFR in 2019: large (> 128), medium (64–127), and small (≤ 63).

^†^Predominant official language used in the CF clinic.

^‡^West includes provinces west of Ontario: Manitoba, Saskatchewan, Alberta, and British Columbia; East includes provinces east of Quebec: Nova Scotia, New Brunswick, and Newfoundland.

^§^Non-White includes Asian, Black, First Nation People, Hispanic, South Asian, other, two or more races, and unknown.

**Table 2:** Data completeness and accuracy.

Variable	Correct # (%)	Incorrect # (%)	Records validated #	Missing records #	Records that could not be validated #	Total records #
Demographic variables						
Date of birth	208 (100%)	0 (0%)	208	0	0	208
Sex	208 (100%)	0 (0%)	208	0	0	208
Race	179 (99.4%)	1 (0.6%)	180	0	28	208
Date of diagnosis	144 (92.9%)	11 (7.1%)	155	2	51	208
CFTR variant^∗^	402 (99.3%)	3 (0.7%)	405	3	8	416
Transplants						
Transplant date	36 (92.3%)	3 (7.7%)	39	0	1	40
Transplant organ	39 (100%)	0 (0%)	39	0	1	40
Clinical measurements						
ppFEV_1_	564 (97.4%)	15 (2.6%)	579	110	4	693
Height (cm)	663 (97.5%)	17 (2.5%)	680	3	10	693
Weight (kg)	659 (97.3%)	18 (2.7%)	677	7	9	693
Hospitalizations						
Primary reason for hospitalization	91 (95.8%)	4 (4.2%)	95	1	1	97
Hospitalization start and end dates	189 (98.4%)	3 (1.6%)	192	0	2	194
CF treatments and medications						
Inhaled antibiotics^†^	989 (98.1%)	19 (1.9%)	1008	0	32	1040
Mucolytics^§^	396 (96.6%)	14 (3.4%)	410	0	6	416
CFTR modulator	27 (100%)	0 (0%)	27	0	1	28
CFTR modulator start date	27 (100%)	0 (0%)	27	0	1	28
Pancreatic enzymes	191 (92.7%)	15 (7.3%)	206	0	2	208
Azithromycin	194 (95.1%)	10 (4.9%)	204	0	4	208
Complications						
Pancreatic insufficiency	206 (99.0%)	2 (1%)	208	0	0	208
CF-related diabetes	203 (98.1%)	4 (1.9%)	207	0	1	208
ABPA	203 (99.0%)	2 (1%)	205	0	3	208
Liver cirrhosis/portal hypertension	204 (99.5%)	1 (0.5%)	205	0	3	208
Respiratory infections						
*Pseudomonas aeruginosa*	196 (97.0%)	6 (3%)	202	0	6	208
MRSA	200 (99.0%)	2 (1%)	202	0	6	208
*Staphylococcus aureus*	200 (99.0%)	2 (1%)	202	0	6	208
*Stenotrophomonas maltophilia*	198 (98.5%)	3 (1.5%)	201	0	7	208
Burkholderia cepacia complex (any)	197 (97.5%)	5 (2.5%)	202	0	6	208
*Mycobacterial* species (any)	73 (98.6%)	1 (1.4%)	74	0	5	79

*Note:* ppFEV_1_: FEV_1_ percent predicted.

Abbreviations: ABPA = allergic bronchopulmonary aspergillosis; MRSA = methicillin-resistant *Staphylococcus aureus*.

^∗^The CCFR database allows the capture of 3 CFTR variants. A “correct” determination was given if both variants listed in the “Mutation 1” and “Mutation 2” fields were validated as correct.

^†^Inhaled antibiotics include the following treatments: aztreonam nebulized treatments, inhaled tobramycin treatments (including Podhaler and other solutions), and colistin nebulized treatments.

^§^Mucolytics include the following treatments: hypertonic saline and DNase.

## Data Availability

The data that support the findings of this study are available on request from the corresponding author. The data are not publicly available due to privacy or ethical restrictions.

## Supporting Information

An Appendix with the data validation program database template that was used for the validation by sites is included.

## References

[1] (2023). *The 2022 Candian Cystic Fibrosis Registry Anuual Data Report*.

[2] Stephenson A. L., Swaleh S., Sykes J. (2023). Contemporary Cystic Fibrosis Incidence Rates in Canada and the United States. *Journal of Cystic Fibrosis*.

[3] (1978). *Report on Survival Studies of Patients with Cystic Fibrosis*.

[4] Petruzziello-Pellegrini T. N., Jeanneret A., Montgomery M., Rivard G., Tullis E., Cantin A. M. (2018). Cystic Fibrosis in Canada: A Historical Perspective. *Canadian Journal of Respiratory, Critical Care, and Sleep Medicine*.

[5] Sykes J., Stanojevic S., Ma X. (2024). Exploring Reduced Pulmonary Exacerbation Rates in Cystic Fibrosis: A Pandemic or Modulator Effect?. *Journal of Cystic Fibrosis*.

[6] Application Form for Accessing Record-Level Canadian CF Registry Data. https://www.cysticfibrosis.ca/uploads/Application%20form%20for%20accessing%20record-level%20CF%20Canada%20Registry%20data.pdf.

[7] Knapp E. A., Fink A. K., Goss C. H. (2016). The Cystic Fibrosis Foundation Patient Registry. Design and Methods of a National Observational Disease Registry. *Annals of the American Thoracic Society*.

[8] Stern M., Wiedemann B., Wenzlaff P. (2008). From Registry to Quality Management: the German Cystic Fibrosis Quality Assessment Project 1995-2006. *European Respiratory Journal*.

[9] Stern M., Niemann N., Wiedemann B., Wenzlaff P. (2011). Benchmarking Improves Quality in Cystic Fibrosis Care: a Pilot Project Involving 12 Centres. *International Journal for Quality in Health Care*.

[10] Corey M., McLaughlin F. J., Williams M., Levison H. (1988). A Comparison of Survival, Growth, and Pulmonary Function in Patients with Cystic Fibrosis in Boston and Toronto. *Journal of Clinical Epidemiology*.

[11] Burgel P. R., Goss C. H. (2021). COVID-19 Outcomes in People with Cystic Fibrosis. *Current Opinion in Pulmonary Medicine*.

[12] Martin B., Schechter M. S., Jaffe A., Cooper P., Bell S. C., Ranganathan S. (2012). Comparison of the US and Australian Cystic Fibrosis Registries: the Impact of Newborn Screening. *Pediatrics*.

[13] Goss C. H., Macneill S. J., Quinton H. (2012). Children and Young Adults in the US Have Improved Lung Function Compared to the UK. *Pediatric Pulmonology*.

[14] Wolf L., Usemann J., Collaud E. (2024). Data Accuracy, Consistency and Completeness of the National Swiss Cystic Fibrosis Patient Registry: Lessons from an ECFSPR Data Quality Project. *Journal of Cystic Fibrosis*.

[15] Stanojevic S., Vukovojac K., Sykes J., Ratjen F., Tullis E., Stephenson A. L. (2021). Projecting the Impact of Delayed Access to Elexacaftor/tezacaftor/ivacaftor for People with Cystic Fibrosis. *Journal of Cystic Fibrosis*.

[16] Arya R. S., Sharpe I., Cheng S. (2024). 699 Rates of Emerging Non-pulmonary Complications in Adults with Cystic Fibrosis and in the General Population. *Journal of Cystic Fibrosis*.

[17] Laflamme O. D., Johnson N., Steele K. (2024). Socioeconomic Burden of Cystic Fibrosis in Canada. *BMJ Open Respiratory Research*.

[18] Zolin A., Adamoli A., Bakkeheim E. (2024). ECFSPR Annual Report 2022. https://www.ecfs.eu/sites/default/files/Annual%20Report_2022_ECFSPR_20240603.pdf.

[19] Cromwell E. A., Ostrenga J. S., Todd J. V. (2023). Cystic Fibrosis Prevalence in the United States and Participation in the Cystic Fibrosis Foundation Patient Registry in 2020. *Journal of Cystic Fibrosis*.

[20] Pellen N., Gueganton L., Pougheon Bertrand D., Rault G. (2018). Lessons from the On-Site Quality Audit of Data Transmitted to the French Cystic Fibrosis Registry. *Orphanet Journal of Rare Diseases*.

[21] Stephenson A., Sykes J., Stanojevic S. (2017). Survival Comparison of Patients with Cystic Fibrosis in Canada and the United States: A Population-Based Cohort Study. *Annals of Internal Medicine*.

[22] Goss C., Sykes J., Stanojevic S. (2018). Comparison of Nutrition and Lung Function Outcomes in Patients with Cystic Fibrosis Living in Canada and the United States. *American Journal of Respiratory and Critical Care Medicine*.

[23] Ahern S., Rusekaite R., Dean J. (2019). Emerging Registry Uses Requires Adapatable Systems: Reinventing the Australian Cystic Fibrosis Registry. *The Australian Cystic Fibrosis Data Registry*.

[24] Polubriaginof F. C. G., Ryan P., Salmasian H. (2019). Challenges with Quality of Race and Ethnicity Data in Observational Databases. *Journal of the American Medical Informatics Association*.

[25] Cerdeña J. P., Grubbs V., Non A. L. (2022). Genomic Supremacy: the Harm of Conflating Genetic Ancestry and Race. *Human Genomics*.

[26] Routen A., Akbari A., Banerjee A. (2022). Strategies to Record and Use Ethnicity Information in Routine Health Data. *Nature Medicine*.

[27] Blustein J. (1994). The Reliability of Racial Classifications in Hospital Discharge Abstract Data. *American Journal of Public Health*.

[28] Gomez S., Glaser S. (2006). Misclassification of Race/ethnicity in a Population-Based Cancer Registry (United States). *Cancer Causes & Control*.

[29] Hamilton N. S., Edelman D., Weinberger M., Jackson G. L. (2009). Concordance between Self-Reported Race/ethnicity and that Recorded in a Veteran Affairs Electronic Medical Record. *North Carolina Medical Journal*.

[30] Nong P., Raj M., Creary M., Kardia S. L. R., Platt J. E. (2020). Patient-reported Experiences of Discrimination in the US Health Care System. *JAMA Network Open*.

[31] Holland R., Stewart H., Cheng S. Y., Schroeder M., Stanojevic S. (2025). Disparities in Outcomes by Race and Ethnicity in the Canadian Cystic Fibrosis Population. *Journal of Cystic Fibrosis*.

