# Supplementary material for: Validating the Data Completeness and Accuracy of the Canadian Cystic Fibrosis Registry
Source: Can Respir J. 2025 Jul 1;2025:8893074. doi: 10.1155/carj/8893074 (PMC12259333; doi:10.1155/carj/8893074)
Supplement: Supporting Information — Additional supporting information can be found online in the Supporting Information section. [file 8893074.f1.docx]

**Appendix A: Data Validation Program Database Template**

Consent

| **CCFR ID** | **Variable** | **CCFR Value** | **Result**  Correct  Incorrect  Missing  Can’t validate | **If incorrect,**  **correct value from medical records** | **Notes** |
| --- | --- | --- | --- | --- | --- |
|  | Consent | Yes |  |  |  |

Patient Data

| **CCFR ID** | **Variable** | **CCFR Value** | **Result**  Correct  Incorrect  Missing  Can’t validate | **If incorrect,**  **correct value from medical records** | **Notes** |
| --- | --- | --- | --- | --- | --- |
|  | First name | Character |  |  |  |
|  | Last name | Character |  |  |  |
|  | Date of birth | Date |  |  |  |
|  | Date of diagnosis | Date |  |  |  |
|  | Date of death | Date |  |  |  |
|  | Mutation 1 | Character |  |  |  |
|  | Mutation 2 | Character |  |  |  |
|  | Race | Caucasian  Asian  Black  First Nations People  South Asian  Hispanic  Other  Two or more races  Unknown |  |  |  |
|  | Sex | Male  Female |  |  |  |
|  | Postal code | Character |  |  |  |
|  | Pancreatic status | Sufficient  Insufficient |  |  |  |
|  | CF-related diabetes | Yes  No |  |  |  |

Transplant Data

| **CCFR ID** | **Variable** | **CCFR Value** | **Result**  Correct  Incorrect  Missing  Can’t validate | **If incorrect,**  **correct value from medical records** | **Notes** |
| --- | --- | --- | --- | --- | --- |
|  | Transplant status date 1 | Date |  |  |  |
|  | Transplant status 1 | Actively listed  Received a transplant |  |  |  |
|  | Organ 1 | Heart  Lung  Liver  Kidney  Pancreas  Other |  |  |  |
|  | … |  |  |  |  |
|  | Transplant status date *n* | Date |  |  |  |
|  | Transplant status *n* | Actively listed  Received a transplant |  |  |  |
|  | Organ *n* | Heart  Lung  Liver  Kidney  Pancreas  Other |  |  |  |

Annual Data (2019)

| **CCFR ID** | **Variable** | **CCFR Value** | **Result**  Correct  Incorrect  Missing  Can’t validate | **If incorrect,**  **correct value from medical records** | **Notes** |
| --- | --- | --- | --- | --- | --- |
|  | Exam date 1 | Date |  |  |  |
|  | FEV1 1 | Numeric |  |  |  |
|  | Height cm 1 | Numeric |  |  |  |
|  | Weight kg 1 | Numeric |  |  |  |
|  | … |  |  |  |  |
|  | Exam date *n* | Date |  |  |  |
|  | FEV1 *n* | Numeric |  |  |  |
|  | Height cm *n* | Numeric |  |  |  |
|  | Weight kg *n* | Numeric |  |  |  |
|  | Hospitalization start date 1 | Date |  |  |  |
|  | Hospitalization end date 1 | Date |  |  |  |
|  | Primary reason for hospitalization 1 | Diabetes  DIOS  G-tube insertion  G-tube removal  Influenza (any)  Kidney stones  Liver disease  Massive hemoptysis  Mental health reasons  New diagnosis of CF  Obstetrical  Other GI disease  Pancreatitis  Pneumothorax  P. aeruginosa eradication  Pulmonary exacerbation  Sinus surgery  Transplant-related  Other |  |  |  |
|  | … |  |  |  |  |
|  | Hospitalization start date *n* | Date |  |  |  |
|  | Hospitalization end date *n* | Date |  |  |  |
|  |  |  |  |  |  |
|  | Primary reason for hospitalization *n* | Diabetes  DIOS  G-tube insertion  G-tube removal  Influenza (any)  Kidney stones  Liver disease  Massive hemoptysis  Mental health reasons  New diagnosis of CF  Obstetrical  Other GI disease  Pancreatitis  Pneumothorax  P. aeruginosa eradication  Pulmonary exacerbation  Sinus surgery  Transplant-related  Other |  |  |  |
|  | Home IV start date 1 | Date |  |  |  |
|  | Home IV end date 1 | Date |  |  |  |
|  | … |  |  |  |  |
|  | Home IV start date *n* | Date |  |  |  |
|  | Home IV end date *n* | Date |  |  |  |
|  | CFTR modulator name 1 | Kalydeco  Orkambi  Symdeko  Trikafta  Other |  |  |  |
|  | CFTR modulator start date 1 | Date |  |  |  |
|  | CFTR modulator end date 1 | Date |  |  |  |
|  | … |  |  |  |  |
|  | CFTR modulator name *n* | Kalydeco  Orkambi  Symdeko  Trikafta  Other |  |  |  |
|  | CFTR modulator start date *n* | Date |  |  |  |
|  | CFTR modulator end date *n* | Date |  |  |  |
|  | Azithromycin | Yes/No |  |  |  |
|  | Hypertonic saline | Yes/No |  |  |  |
|  | Pulmozyme/DNase | Yes/No |  |  |  |
|  | Pancreatic enzyme | Yes/No |  |  |  |
|  | Cayston/Aztreonam nebulized treatments | Yes/No |  |  |  |
|  |  |  |  |  |  |
|  | TOBI nebulized treatments | Yes/No |  |  |  |
|  | TOBI podhaler | Yes/No |  |  |  |
|  | Collistin nebulized treatments | Yes/No |  |  |  |
|  | Inhaled Levofloxacin | Yes/No |  |  |  |
|  | Other tobramycin solution/Nebcin | Yes/No |  |  |  |
|  | Vancomycin nebulized treatments | Yes/No |  |  |  |
|  | ABPA | Yes/No |  |  |  |
|  | Liver cirrhosis/portal hypertension | Yes/No |  |  |  |
|  | Pseudomonas aeruginosa | Yes/No |  |  |  |
|  | MRSA | Yes/No |  |  |  |
|  | Staphylococcus aureus | Yes/No |  |  |  |
|  | Stenotrophomonas maltophilia | Yes/No |  |  |  |
|  | Burkholderia cepacia complex (any) | Yes/No |  |  |  |
|  | Mycobacterial species (any) | Yes/No |  |  |  |
